# International Clinics

**Published:** 1903-03

**Authors:** 


INTERNATIONAL CLINICS.—A quarterly of illustrated clinical lectures and especially prepared articles on medicine, neurology, surgery, therapeutics, obstetrics, pediatrics, pathology, dermatology, diseases of the eye, ear, nose and throat, and other topics of interest to students and practitioners by leading members of the medical profession throughout the world. Edited by Henry W. Cattell, A. M., M. D., Philadelphia, U. S. A., with the collaboration of John B. Murphy, M. D., Chicago; Alexander D. Blackader, M. D., Montreal; H. C. Wood, M. D., Philadelphia; T. M. Rotch, M. D., Boston; E. Landolt, M. D., Paris; Thomas G. Morton, M. D., Philadelphia; James J. Walsh, M. D., New York; J. W. Ballantyne, M. D., Edinburgh, and John Harold, M. D., London, with regular correspondents in Montreal, London, Paris, Leipsic and Vienna. J. B. Lippincott Company, Philadelphia and London. Cloth, $2.00. Volume I, twelfth series, 84 illustrations, 3 colored plates.

This volume is begun with two biographical sketches, one of Dr. Weir Mitchell, of Philadelphia, and the other of Dr. John A. Wyeth, of New York. These men are still living, but are coasting along leaving the wealth of their careers to encourage those who follow.

This book is especially well illustrated. All of the twenty articles which it contains are attractively gotten up, and were prepared for the clinics. The latter 124 pages are devoted to the progress of medicine for 1901. This department is under the charge of Dr. E. W. Watson. T. J. B.

